# Molecular Diagnostic Review of Diffuse Large B-Cell Lymphoma and Its Tumor Microenvironment

**DOI:** 10.3390/diagnostics12051087

**Published:** 2022-04-27

**Authors:** Robert Ta, David Yang, Christian Hirt, Thomas Drago, Richard Flavin

**Affiliations:** 1 Massachusetts General Hospital, Harvard Medical School, Boston, MA 02114, USA; rta1@mgh.harvard.edu; 2 Beth Israel Deaconess Medical Center, Harvard Medical School, Boston, MA 02215, USA; diyang@bidmc.harvard.edu (D.Y.); chirt@bidmc.harvard.edu (C.H.); 3 Department of Histopathology, St. James’s Hospital and Trinity College, D08 NHY1 Dublin, Ireland; dragot@tcd.ie

**Keywords:** molecular, diagnostics, diffuse large B-cell lymphoma, cell-of-origin, immunohistochemistry, gene expression profiling, cytogenetics/FISH, minimum criteria for diagnosis

## Abstract

Diffuse large B-cell lymphoma (DLBCL) is the most common non-Hodgkin lymphoma. It is a clinically and morphologically heterogeneous entity that has continued to resist complete subtyping. Molecular subtyping efforts emerged in earnest with the advent of gene expression profiling (GEP). This molecular subtyping approach has continued to evolve simultaneously with others including immunohistochemistry and more modern genomic approaches. Recently, the veritable explosion of genomic data availability and evolving computational methodologies have provided additional avenues, by which further understanding and subclassification of DBLCLs is possible. The goal of this review is to provide a historical overview of the major classification timepoints in the molecular subtyping of DLBCL, from gene expression profiling to present day understanding.

## 1. Introduction

Diffuse large B-cell lymphoma (DLBCL) is the most common non-Hodgkin lymphoma in the western hemisphere [1]. Diagnosis and subtyping of DLBCL has come far to date, from just requiring morphological assessment based on a single H & E slide to now, where numerous ancillary tests are a prerequisite, including immunohistochemistry, cytogenetics, flow cytometry, and molecular testing. Whilst these technologies continue to allow refinements in diagnostic subtyping, the highly heterogeneous nature of the disease continues to evade full subtyping efforts. Furthermore, continued efforts to understand the underlying molecular pathophysiology of the disease are needed, given the propensity of the disease to relapse beyond standard and even precision therapies. The aim of this review is to provide an overview of the historical and current state of DLBCL molecular subtyping. Furthermore, we review recent reports that aid in the understanding of the pathophysiology of DLBCL and also explore evidence of how this entity interacts with its surrounding microenvironment.

## 2. Gene Expression Profiling

**Figure 1:**
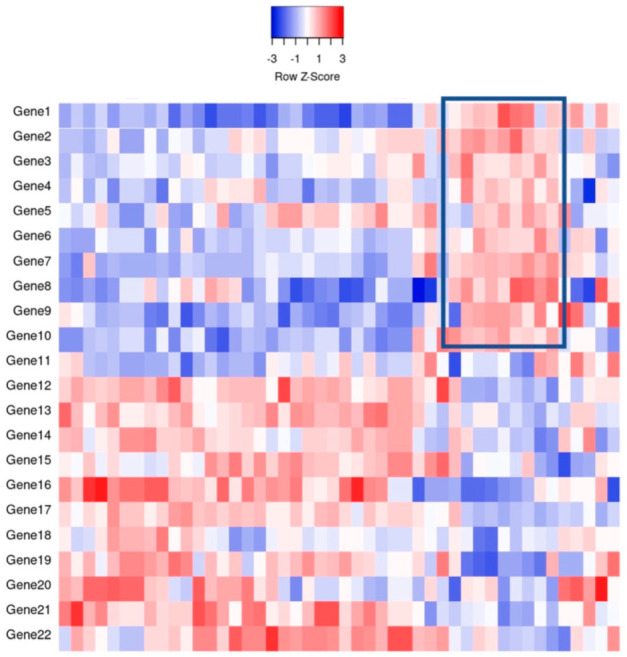
An example of gene expression profiling comparing the relative gene expression levels and grouping of cases by clusters. For example, the indicated boxed area indicates that Genes 1–10 have a relative increase in expression, thereby potentially clustering cases together for classification purposes.

It was not long ago that the diagnosis of liquid tumors rested on morphology, immunohistochemical analyses, and cytogenetics. The original diffuse large B-cell lymphoma (DLBCL) molecular subclassification followed the advent of DNA microarrays, a technology that allows the analysis of thousands of expressed genes simultaneously; see Figure 1 for a theoretical example [2]. This DNA microarray-based technology allowed for transcriptional gene pattern expression (e.g., Lymphochip by Alizadeh et al. [3]) analysis under defined conditions that delineated the seminal molecular study classifying a liquid cancer DLBCL into the so-called cell of origin (COO) subtypes [4]. This accomplishment resulted in the subtyping of approximately 80–85% of all DLBCL cases and importantly showed subtype prognostic values that were greater than that of the standard clinical predictor, the International Prognostic Index (IPI) [4]. In addition, this critical work paved the way for immunohistochemical (IHC) determination of COO.

Although the study population was small by current standards, with imperfect subtyping, the classification was adopted and continues today as the standard of diagnostic care, recognized within the World Health Organization’s Revised Fourth Edition of the Classification of Tumors of the Hematopoietic and Lymphoid Tissues [5] as germinal center B-cell like (GCB) and activated B-cell like (ABC), with the remainder left as unclassified. Novelty is never without controversy as other approaches, including an unbiased a priori approach which used supervised machine learning to analyze GEP, did not find molecular correlates of COO to be independently prognostic [6]. Nevertheless, the same group reported differing GEP signatures that predicted response to CHOP chemotherapy (cyclophosphamide, doxorubicin hydrochloride, vincristine, and prednisone). Indeed, in that vein, groups like Rosenwald et al. [7] reported cell specific and non-cell specific genetic signatures that differed depending on response to standard chemotherapies. Regardless of molecular prognostication and subtyping, molecular investigation was certain to provide increasing identification of precision therapeutic targets based on biochemical pathways [4,6,7,8].

Despite the reasonable success of standard CHOP and R-CHOP, and the growing number of precision therapies, heterogeneity remained an issue, as evidenced by inconsistent treatment responses and relapses despite COO subtyping. Although gaining favor and resolving power, it was thought perhaps that the transcriptionally based GEP was an inadequate representation of underlying aberrant genetic programming (e.g., a DNA repair protein with single nucleotide polymorphism). However, practically, other technical hurdles, such as the logistical necessity for fresh tissue specimens, stood in the way of immediate clinical adoption of GEP in the subcategorization of DLBCLs. Initially, this was a difficult step to overcome, however several novel testing options were eventually able to surmount this issue, including Nanostring [9], HTG [10], and Roche [11]. These options allowed the use of formalin fixed paraffin embedded (FFPE) tissues, which was in keeping with standard pathologic workflow and also allowed for an increase in analytical case numbers to be studied.

Meanwhile, through whole exome sequencing and transcriptome sequencing, oncogenic drivers of DLBCL were mapped [12]. These findings reported that the most unfavorable prognoses were DLBCL cases with *MYC* aberrations along with MYC IHC over-expression. Other studies using mouse modelling also supported the molecular ideas emerging from GEP, showing oncogenic driver mutations in genes such as *EZH2* and *MYD88* that promoted lymphoma development [13,14]. Simultaneously on the molecular subclassification side, other analytic hurdles, such as setup and integration of the multiple platforms (e.g., whole-exome sequencing and RNA sequencing), were required to adequately identify different types of genetic aberrations, including mutations, translocations, and copy number alterations [15,16].

**Figure 2:**
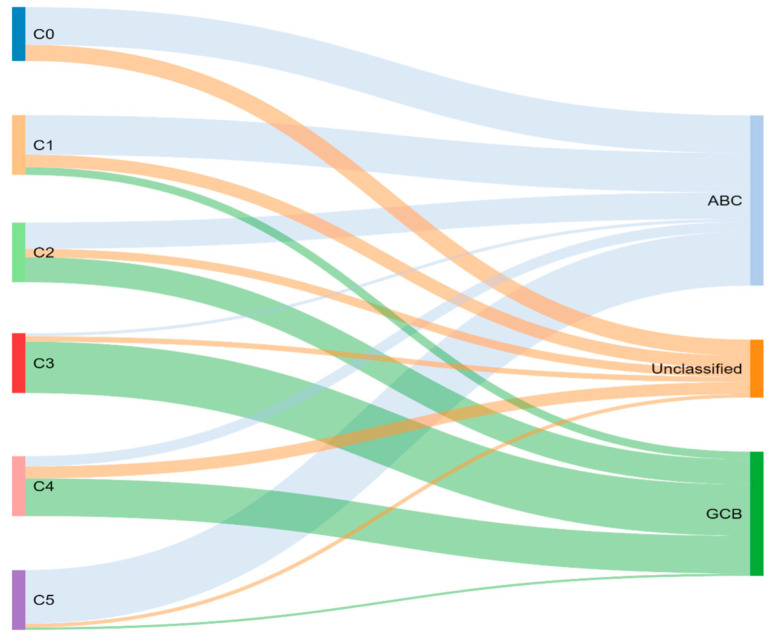
Chapuy and colleagues’ genetic cluster classification of DLBCL subtype, as compared to cell of origin. C0–C5 = cluster 0 to cluster 5.

Through previously mentioned genomic methods, Chapuy et al. [15] utilized clustering analytic methods to identify low-frequency alterations, captured recurrent mutations, somatic copy number alterations, and structural variants, and created five differential genetic signatures to further subtype DLBCL (Figure 2). C1 was ABC-related, associated with *NOTCH2* mutations and favorable outcome; C2 was unrelated to GCB and ABC, had frequent biallelic *TP53* inactivation, *CDKN2A* deletion, and poor outcomes; C3 was GCB-related, associated with *BCL2* translocation, *PTEN* aberrations, epigenetic modifiers (*KMT2D*, *CREBBP*, and *EZH2*) and unfavorable outcome; C4 was GCB-related, with BCR–PI3K, NF-κB, or RAS–JAK signal transducer, was an activator of transcription (BRAF and STAT3) pathway aberrations, histone gene mutations, cluster of differentiation proteins associated with immune evasion (CD83, CD70, and CD58), and had favorable outcomes; C5 was ABC related, gained *BCL2*, *MYD88*L265P, *CD79B*, *PIM1*, and *PRDM1* mutations, and had unfavorable outcome. The prognostic capability of the subtypes was also independent of the clinical gold standard IPI [15]. 

**Figure 3:**
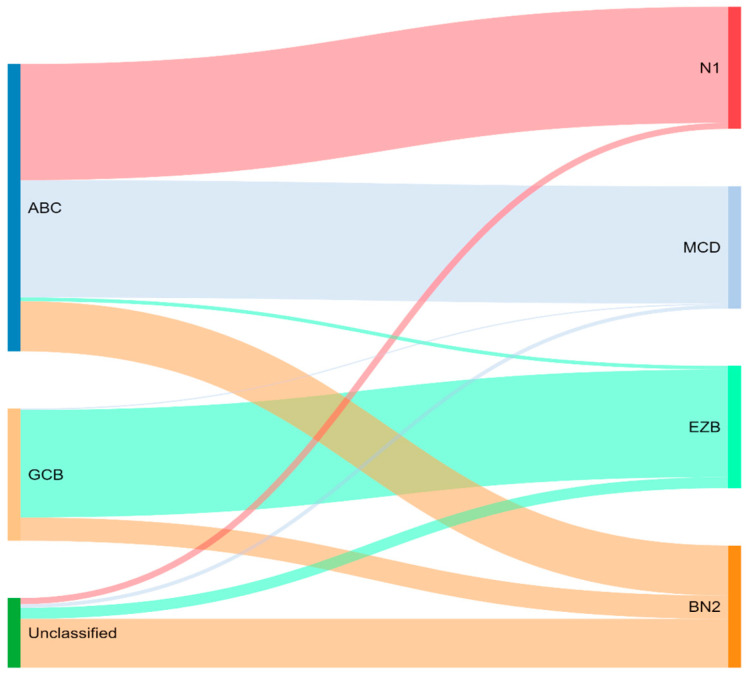
Schmitz and colleagues’ genetic classification of DLBCL subtype as compared to cell of origin. ABC = activated B-cell, GCB = germinal-center B-cell, N1 = notch 1, MCD = MYD88L265P, CD79B co-mutation, EZB = EZH2 mutation or BCL2 translocation, and BN2 = Notch 2.

Concurrently, Schmitz et al. [16] simultaneously characterized four DLBCL subsets via their GenClass algorithm, termed MCD (MYD88L265P, CD79B co-mutation), BN2 (BCL6 fusions or NOTCH2 mutation), N1 (NOTCH1 mutations), or EZB (EZH2 mutation or BCL2 translocation), based on a more homogenous set of genomic aberrations (Figure 3). Interestingly, they were able to identify precision targets within the high-risk subtypes, showing, for example, that MCD could be more responsive to ibrutinib secondary to constitutive BCR signaling [16].

To compare, the Chapuy C1, C3, and C5 clusters overlapped with the Schmitz GenClass BN2, EZB, and MCD groups, respectively. The Chapuy C2 and C4 subtypings did not overlap with any of the other Schmitz subtypings. This non-concordance was perhaps thought secondary to differences in bioinformatic analytic approaches.

For the last two decades, R-CHOP has been the standard of treatment in previously untreated DLBCL. With the molecular subtyping of DLBCL, it was proposed that a subtype specific treatment could improve response rates specially for patients who do not achieve complete remission or develop disease relapse (around 40% treated with R-CHOP) [17]. Ibrutinib, a first-in-class oral covalent inhibitor of Bruton’s tyrosine kinase (BTK) showed some preferential activity in ABC DLBCL [18]. In a randomized multicenter study [19], the goal was to determine if addition of ibrutinib would improve efficacy of R-CHOP in ABC DLBCL. Interestingly, the addition of ibrutinib to R-CHOP improved event-free survival and overall survival in patients younger than 60 years. Unfortunately, older patients (>60 years of age) had increased serious adverse effects with ibrutinib plus R-CHOP. Of note, molecular subtyping increased median time to diagnosis by 27 days, which may have excluded patients necessitating immediate treatment. This study shows the potential of subtype specific treatment as well the need for reasonable turnaround diagnostic times if integrating molecular subtyping. Subsequently, the PHOENIX trial continued to demonstrate superior outcomes in younger patients treated with ibrutinib and R-CHOP, but also better overall survival in specific molecular subsets including the MCD and N1 subgroups compared to R-CHOP alone [20]. Landsburg and colleagues found that ibrutinib monotherapy had a 60% response rate in relapsed/refractory patients with a non-germinal center, and MYC and BCL2 double expressor phenotype [21]. 

The ROBUST study is a phase 3 clinical trial that compared the addition of lenalidomide to rituximab plus cyclophosphamide, doxorubicin, vincristine, and prednisone (R-CHOP) therapy with R-CHOP therapy alone for treatment of activated B-cell-like (ABC) subtype of diffuse large B-cell lymphoma (DLBCL). ABC-type DLBCL has traditionally been shown to resist typical R-CHOP therapy, however, emerging phase 2 studies are demonstrating promise of the addition of lenalidomide to R-CHOP (R2-CHOP) in ABC-type therapy. The primary end point of the study was progression-free survival (PFS) of participants receiving R2-CHOP, compared to those receiving R-CHOP only. Although PFS was not met (hazard ratio 0.85), the median PFS was not reached for either group. PFS tended to favor R2-CHOP over placebo group in patients with higher-risk disease, but adverse events of R2-CHOP compared to placebo were neutropenia (60% vs. 48%), anemia (22% vs. 14%), thrombocytopenia (17% vs. 11%), and leukopenia (14% vs. 15%). Of note, ROBUST was the first phase 3 study to highlight biomarker identification of ABC patients and was able to demonstrate a consistent safety profile of R2-CHOP.

Initial hopes were high that the development of molecular GEP would have significant effects on prognostic DLBCL classification, leading to therapeutic tailoring. With the advent of GEP, studies naturally attempted retrospective gene expression profiling analyses on their DLBCL cohorts, unfortunately, with conflicting results. Davies et al. (REMoDL-B) were the first to show that GEP for therapeutic assignment was possible prospectively [22]. However, their randomized phase 3 clinical trial results were disappointing, reporting that the addition of bortezomib to suppress the GEP apparently increased NF-kB gene expression to standard R-CHOP, and failed to improve survival in ABC DLBCLs. While the trial itself was not without criticism, the study lent some doubt as to whether GEP classification was a prognostic and therapeutic breakthrough.

## 3. Immunohistochemistry

While the clinical and translational utility of multi-platform genomic analyses were clear, especially given the increasing interest in personalized medicine, the practicality of implementing complex genomic workflows into daily clinical practice with unclear remuneration and logistical difficulties coordinating fresh tissue samples was evident. This drove interest in immunohistochemical (IHC) staining to both confirm and temporarily bypass genomic efforts. Concurrently, with genomic studies, the utilization of standard immunohistochemistry was investigated.

**Figure 4:**
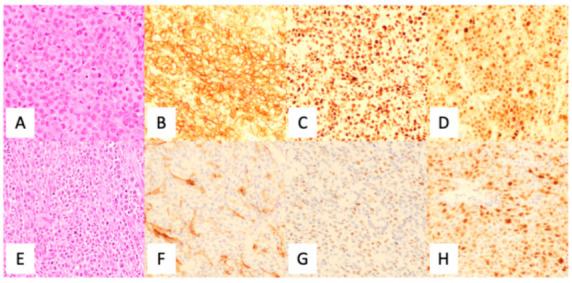
Utilization of the Hans algorithm to determine cell of origin in the diagnosis of diffuse large B-cell lymphoma. (**A**) H & E, (**B**) CD10 positive (membranous), (**C**) BCL6 positive (nuclear), and (**D**) MUM1 positive (nuclear) indicating a germinal center origin. (**E**) H & E (**F**) CD10 negative (non-specific stromal staining), (**G**) BCL6 negative, and (**H**) MUM1 positive (nuclear) demonstrating non-germinal center origin. CD20 is positive in large cells (not shown). Credit: Case from Massachusetts General Hospital.

The current diagnosis of DLBCL was primarily accomplished by utilizing immunohistochemistry (IHC) analysis, using a standard panel of antibodies following morphological review as per the current 2017 WHO Classification of Tumors of the Hematopoietic and Lymphoid Tissues [5]. Although there were various algorithms proposed (see Table 1), the current WHO Classification supports the utilization of the Hans criteria classification for COO subtyping (Figure 4). Although IHC has long been of standard use in the diagnosis of DLBCL, it is known that the Hans criteria itself has approximately an 80% concordance with GEP derived GC-DLBCL and ABC-DLBCL differentiation [23]. 

Clearly, the major advantage of IHC was its ability to be performed on formalin-fixed paraffin-embedded tissue (FFPET). This pathology-based workflow has long been established with relatively quick turnaround and provides a fairly robust subtyping. However, IHC diagnostic accuracy is challenged by GEP as staining interpretation of FFPE can show artifacts, variations in staining strength, and inter-observer variations in histopathologic diagnosis. 

Nevertheless, as GEP and multi-genomic platform analytic efforts continued, IHC was also employed to validate genomic findings [27]. IHC was directed towards finding localizing cell types and assessing numerous types of prognostic variables, including tumor infiltrating lymphocytes [28], tumor microenvironment proteins [29], tumor suppressor expression [30], SPARC positive macrophages [31], immune checkpoints modifiers [32], and others. However, as IHC can be variably interpretable and further restricted to phenotypic appearance, the prognostics derived from such techniques can be controversial. As costs began to decrease and access to genomic data and novel computational tools concomitantly increased, this has allowed for deeper and more precise molecular analyses that can help elucidate the genetic programming of both tumor cell and microenvironment [27].

Another vital use for immunohistochemistry is the prognostic value of double expressor DLBCLs, which indicates poorer patient outcomes. Double expressors are defined as having expression of both MYC ≥ 40% and BCL2 ≥ 50% within lymphoma cells. Initially studied by Green et al. [33], they established a correlation with the double-hit score, which was a strong predictor of patient outcomes, including poor performance status, advanced-stage disease, higher Ki67 proliferative index, and inferior complete response to R-CHOP chemotherapy. Other studies have also supported this, with demonstration of reduced progression free survival or overall survival [34,35,36].

## 4. Genetic Classification

With IHC, cytogenetics, and fluorescence in-situ hybridization (FISH) already a stalwart presence in the diagnosis of hematopoietic tumors, the molecular movement continued to build in liquid tumors for the improved understanding of molecular pathophysiology, subtyping, and discovery of precision targets. However, DLBCL heterogeneity continued to plague analyses and the ultimate realization of personalized therapy, with some cases being unable to be definitively subtyped into a category. For example, Schmitz et al.’s finding of singular *TET2* gene mutations in a portion of their unclassifiable DLBCL population would suggest subtyping was incomplete.

In one more niche subtyping area, researchers were able to further subtype DLBCL cases into the so called double and triple hit DLBCL categories that were previously cryptic to FISH. Ennishi et al. [37] utilized targeted resequencing, whole-exome sequencing, RNA sequencing, and immunohistochemistry to develop a gene-expression signature (DHITsig) that was able to distinguish HGBL-DH/TH-*BCL2* from GCB-DLBCL. Simultaneously, Sha et al. [38] reported a group termed molecular high grade (MHG) with gene expression signatures between that of DLBCL and Burkitt lymphoma. Both novel aggressive subtypes roughly doubled the number of DLBCL tumors that could be classified as HGBL-DH/TH-*BCL2* based on FISH alone, with DHITsig further demonstrating that at least 19% of HGBL-DH/TH-*BCL2* were cryptic to FISH [39].

Building and unifying prior nosological molecular classifications [15,16], Wright et al. probabilistically defined seven genetic subtypes of DLBCL based on subtype predictor genes (i.e., mutations, copy number alterations, fusions) that showed distinct GEPs, immune microenvironments, and outcomes following immunochemotherapy [40]. This publicly accessible algorithm was termed LymphGen, and more specifically classified tumors via a Bayesian prediction model into seven distinct genetic subtypes (i.e., MCD, N1, A53, BN2, ST2, EZB/MYC+, and EZB/MYC-) with genetic themes and further identification of precision drug targets within each subtype. They studied the effects of inhibiting relevant canonical biochemical pathways found in their analyses via utilization of loss-of-function CRISPR/Cas9 on cell lines modeled after each of the seven subtypes. This allowed further interrogation of the tumor’s natural history and a better understanding of the putative drug effects on the canonical biochemical pathways [40].

Interestingly, some of the LymphGen subtypes had more indolent features. Follicular lymphomas were represented by EZBs, marginal zone by BN2, and ST2 by signatures similar to both nodular lymphocyte predominant Hodgkin’s lymphoma and T cell histiocyte rich large B-cell lymphoma. Notably, the MCD subtypes harbored genetic profiling that hinted at immune escape mechanisms, with many being involved in immune privileged sites [40]. They argue that their results are concordant with the idea that multiple genetic hits (e.g., dysregulation of MHC class I, T, and/or NK cell activation) were required to escape immunosurveillance and allow development within more immune privileged sites [41].

In the realm of double and triple hit lymphomas, they refined the DHIT signature by focusing applicability onto GCB cases with poorer outcomes. They suggest that sequential somatic genetic aberrations occur with EZB-MYC subtypes that underlie evolution to EZB-MYC+, with only 38% of EZB-MYC+ cases being double hit (i.e., 38% cases had MYC abnormality, and 78% with a BCL2 translocation). Ultimately, they showed, in GCB cases without EZB mutations, the DHITsig did not correlate adversely with outcomes [40]. These findings shed more light into the natural history of DLBCL and the histologic phenomenon in which diagnostic evidence of more than one lymphoma was present in the same specimen (e.g., composite [42]) or there was evidence of clonal genomic evolution into another entity [43] (e.g., transformation [44]).

Finally, each subtyping established independent differences in standard R-CHOP treatment response and further identified potential areas in which molecular targets could be amenable to existing precision drugs, such as BTK inhibitors (BN2, MCD, A53), lenalidomide (MCD, BN2), BET inhibitors (MCD, BN2), JAK1 inhibitor (MCD), IRAK4 (MCD), EZH2 inhibitors (EZB with EZH2 mutations), venetoclax, or navitoclax (MCD) [40]. If the LymphGen classification continues to gain speed, it will aid in the future establishment of nosology and clinical trial definition.

### Cytogenetics and/or Fluorescence In-Situ Hybridization

**Figure 5:**
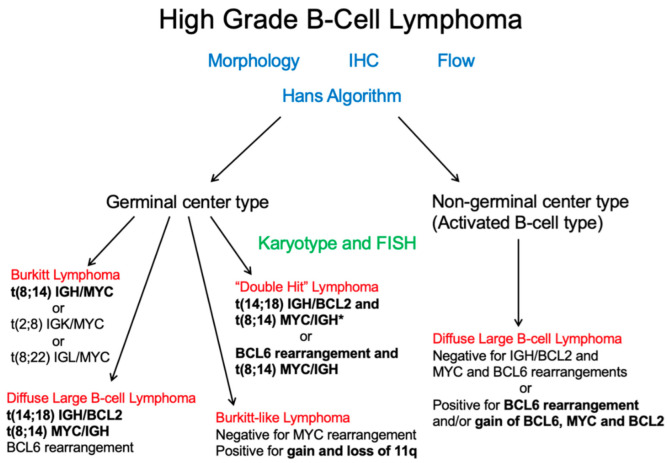
After application of morphologic, immunophenotypic, and the Hans algorithmic assessment, additional cytogenetic analysis can help exclude “double” or “triple” hit lymphomas, a distinct entity as indicated by the 2016 WHO. *Although “double-hit” lymphomas most commonly have an IGH/MYC rearrangement, according to the WHO classification, any MYC rearrangement can occur. Credit: Dr. Christine Bryke, MD at Beth Israel Deaconess Medical Center, Boston, MA.

**Figure 6:**
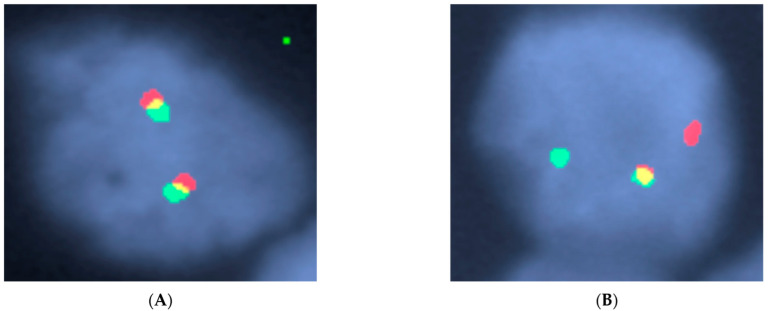
(**A**) Normal MYC break apart probe result with two fusions (two red signals, two green signals). (**B**) MYC FISH break apart probe demonstrating one fusion (one yellow signal) and one broken apart (one green signal and one red signal) indicating a translocation of the MYC gene. This was an example of a high-grade B-cell lymphoma with MYC, BCL2, and BCL6 rearrangements (“triple-hit” lymphoma). Credit to Center for Integrated Diagnostics Laboratory at Massachusetts General Hospital.

Cytogenetics and FISH studies, in addition to identifying abnormalities associated with DLBCL, has the role of excluding more aggressive high-grade lymphomas including double or triple hit lymphomas (Figure 5). These require at least a rearrangement of the MYC gene, BCL6, and/or BCL2 (Figure 6). Historically, FISH for MYC rearrangements on DLBCL was performed in the presence of high-grade morphologic features or high proliferation rate. However, these features do not reliably identify double hit or triple hit lymphomas [45]. A more simplified algorithmic approach was adopted by the Royal College of Pathologists (UK) as outlined in the 2015 lymphoma dataset [46], which recommends testing MYC and BCL2 by FISH. If the cell of origin is of GCB subtype, then MYC FISH +/− BCL2 FISH or BCL2 IHC testing is recommended. Similar guidelines were adopted by the College of American Pathologists (CAP), in which MYC IHC may be helpful in predicting MYC translocations with subsequent FISH confirmation [47].

According to Scott et al. [48], the best method for detecting all high-grade B-cell lymphomas with MYC and BCL2 and/or BCL6 rearrangements (HGBL-DH/TH) among tumors with DLBCL morphology is to screen all DLBCLs for MYC rearrangements. When the result is positive, it should be further tested for BCL2 and BCL6 gene rearrangements. In contrast, FISH testing limited to GCB DLBCLs would decrease FISH testing to half of DLBCLs and would still detect almost all HGBL-DH/TH with BCL2 rearrangements. This method is suitable for MYC/BCL2 HGBL-DH detection, but would miss a significant number of MYC/BCL6 HGBL-DH where the prognostic value is still debated [49,50]. In addition, this approach would fail to identify DLBCLs with isolated MYC rearrangement and ABC/non-GCB phenotype. A major point of the study was to show that selecting DLBCLs with double expressor status and/or COO subtyping results in missing ≈35% of all HGBL-DH.

As a result, Scott et al. [48] demonstrates the impact of various FISH testing strategies to identify HGBL-DH/TH in tumors with DLBCL morphology. FISH testing for MYC, BCL2, and BCL6 should be incorporated in the routine diagnostic workup of all DLBCLs in an integrated approach together with gene expression assays and next-generation sequencing. If this is not cost-effective, another alternative is a two-step method with initial testing for the MYC rearrangement, and to perform FISH for BCL2 and BCL6 if there is a MYC rearrangement. Other screening strategies to limit the costs should be discussed at each institution depending on the local resources and with the knowledge of the limitations of each strategy.

## 5. Microenvironment

While the vast attention of treating DLBCL is focused on the more autonomous phenotypic and functional characteristics of the entity itself, it is relatively easy to overlook the idea of the tumor microenvironment (TME) having any direct or perhaps multi-directional effect on the disease process, especially in liquid tumors. Although the morphologic appearance of DLBCL is that of a single cloned entity effacing its environmental niche, it is thought that other immune and non-immune cells likely play a part in the story. More recently, some groups have begun querying the interactions between DLBCL and its microenvironmental niche, an area in which solid carcinomas have already been well investigated [51,52].

A revisitation of Alizadeh et al. [4] notes that among their GEP COO classification schema, they report an additional bulk GEP signature within the DLBCL milieu consisting of T cells, macrophages, stromal factors, and cytokines with overlapping genetic expression in samples of normal lymph nodes. This is consistent with generally what TMEs are thought to be comprised of, including fibroblasts, endothelial cells, lymphoendothelial cells, tumor associated macrophages, mast cells, lymphocytes, and extracellular matrix/proteins; all, additionally, with active crosstalk [53]. In support of the compositional aspect as well as the idea that the TME can influence the natural history of lymphomas in general, studies have shown TME cell types contribute to crosstalk and, thereby, evolution of liquid tumors and their response to treatment [54,55,56]. 

Lenz et al. [8] reported two microenvironmental gene expression signatures (i.e., stromal-1 and stromal-2) that predicted survival in patients receiving CHOP and R-CHOP chemotherapies for DLBCL. Stromal-1 was associated with improved overall survival and included GEP signatures associated with mesenchymal and macrophage activity. Stromal-2 was associated with poorer overall survival and showed endothelial cell activity signatures along with expression of key angiogenic regulators, leading to increased vasculogenic burden [8]. This data was also seen by Cioronianu et al. [57] and the growing idea then, that TME stroma could be protective, piqued further interest. 

However, as mentioned, the cost of technology and scarcity of fresh tissue greatly hampered research efforts. As such, attempts were made to confirm and perhaps circumvent GEP via flow cytometry and immunohistochemistry/immunofluorescence (IHC/IF) of FFPET by taking strong GEP prognosticators and attempting to confirm their value [28,29,58,59]. Unfortunately, the studies were inconsistent and hence controversial, though secondary to issues inherent to IHC/IF such as staining and inability to capture actual functionality of the TME [59].

With the advent of new technologies, including NanoString, whole-exome and whole-transcriptome sequencing coupled with new deconvolutional computational methods and increasing availability of genomic data, the dissection of TMEs more deeply from bulk to the single-cell level was rendered possible and economically feasible, delivering a surge of interest in the search for novel prognostications and therapeutics based on microenvironmental interactions [60,61].

Recently, Ciavarella et al. [60] analyzed available genomic data in DLBCLs using the CIBERSORT computational methodology to map and discover prognostic variables within the TME. NanoString was utilized to validate the findings, wherein they discovered two profiles that were predictive of survival independent of COO. Specifically, cases with increased myofibroblasts, CD4 positive T-cells, and dendritic cells showed better overall survival when compared with cases showing activated NK and plasma cells. Although, interestingly, Ghorab and colleagues found no association with T-cell mediated immunity and tumor progression [62]. This may speak more to the nature of the tumor heterogeneity. Evaluating the tumor microenvironment may potentially play a significant role in treatment algorithms for patients with relapsed or refractory DLBCL, as highlighted by Solimando et al. [63]. Notably, the integration of the TME with standard COO prognostication improved overall survival prediction [60]. 

Most recently, Kotlov et al. [64] performed a transcriptomic analysis from multiple cohorts by developing functional gene expression signatures of single cells (e.g., fibroblasts, tumor infiltrating lymphocytes and macrophages), cytokines, extracellular matrix, cell proliferation signatures, cell secretion signatures, and canonical cell signaling pathways (e.g., PI3K, NFkB) to ultimately describe four independently prognostic communities of the TME. Further, through mouse modeling, they provided evidence for tumor epigenetic hypermethylation mechanisms and a rationale for the use of DNA hypomethylating agents and extracellular matrix proteins to decrease tumor burden [64].

**Figure 7:**
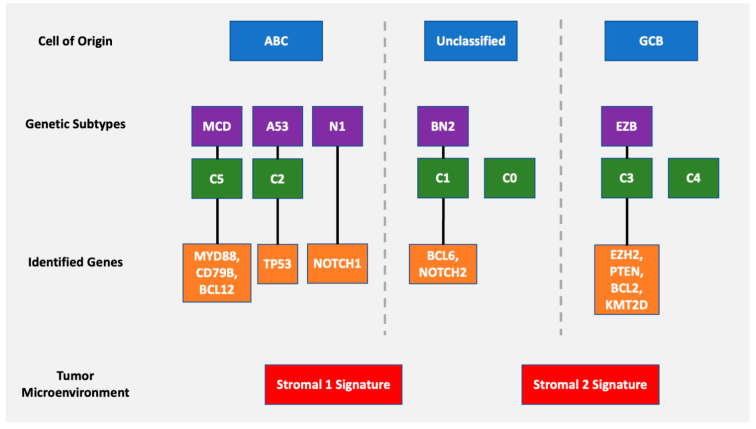
Overview of various DLBCL classification schemes including cell of origin, genetic subtyping with identified genes, and tumor microenvironment.

Given growing evidence that the prognostic power of the TME holds significance independent of current standards including COO and the international prognostic index (IPI), it is an area of clear pathophysiologic importance (Figure 7). Future studies will likely seek to elucidate how the immunopathophysiolgical and extracellular element crosstalk interacts with the seemingly non-autonomous tumor body to affect the natural history of treated and untreated DLBCLs.

## 6. Minimal Diagnostic Criteria

The revised WHO 2017 classification uses the examination of tumor cell morphology and immunophenotype, testing for recurrent chromosomal rearrangements, and integration with clinical and radiologic information, including the disease location and presence of immunosuppression [65]. As discussed, recent studies using genomic, epigenomic, transcriptomic, proteomic, and microenvironmental alterations have additionally expanded the knowledge of DLBCL pathophysiology. These technologies may help to identify new predictive biomarkers and drug targets, allowing for clinical trials and ultimately personalized treatment.

As those technologies remain directly available mostly only in academic centers and are associated with significant costs, rational and methodic approaches for the initial evaluation of DLBCL for community practice pathology still remains the base of any further genomic workup (Table 2) [66]. It is important to mention that there is balance between forcing classification of individual tumors and leaving tumors unclassified because of insufficient classification confidence. Using the classical immunophenotyping, DLBCL can be divided into ABC and GCB subtypes, leaving about 20% unclassified. Subprofiling based on genomic and gene expression markers has allowed profiling of more subgroups which show a differential response to chemotherapy and targeted agents, as reported in Chapuy et al. [15] and Schmitz et al. [16]. Up to 43.4% remain unclassified, and even with recent algorithms incorporating genomic data from multiple analytic platforms, such as the LymphGen [40], up to 32.9% of the cases cannot be classified in one of the defined subgroups, such as MCD, BN2, EZB, ST2, or A53. 

In countries with limited resources, following H & E review, an immunohistochemistry panel with the markers for CD20, CD5, CD21, and Ki67 reaches a correct diagnosis of DLBCL in 83% of the cases [67]. Additionally, to allow detection of EBV-positive DLBCL, all potential cases should be worked up for EBV using in situ hybridization for EBV-encoded small RNA (EBER). The Hans algorithm still remains as one of the cornerstones of an initial DLBCL assessment, without the need for gene expression studies. CD10, BCL6, and IRF4/MUM1 IHCs are necessary to perform the algorithm. Finally, IHC for BCL2 and MYC should be performed to identify double expressor DLBCL, which were shown to be associated with a relatively poor prognosis [68]. Cytogenetic FISH testing for rearrangements of *MYC* and *BCL2* and/or *BCL6*, helps to identify double-hit or triple-hit, high grade B-cell lymphoma with even poorer outcome and the need for more intensive treatment plans [69]. 

After an initial histopathological workup, the specimen could be analyzed using a targeted sequencing panel of a limited set of genes either by sending to a sequencing company or to a central hematopathology laboratory. A more centralized or standardized approach would allow collection of additional data regarding predictive biomarkers, and eventually attribution to clinical trials. 

## 7. Conclusions

The classification of DLBCL has come a long way, and molecular techniques have provided a wealth of information to the underlying pathophysiology, prognostic, diagnostic, and therapeutic implications. Even at the time of writing and publishing this review, there will be undoubtedly be updates, if that is any indication of the speed of development in molecular techniques and novel technology that are able to further enhance our understanding of this heterogenous and diverse lymphoma.

## Figures and Tables

**Table 1:** Various proposed algorithms for cell of origin subtyping. The Hans criteria is currently accepted and adopted by the most recent WHO classification of hematopoietic and lymphoid tissues.

Algorithm	Antibodies	Comments
Hans [23]	CD10, BCL6, MUM1	≥30% staining to be considered positive
Choi [24]	CD10, BCL6, MUM1, GCET, FOXP1	≥80% GCET, FOXP1, MUM1 ≥30% staining for CD10 and BCL6
Muris [25]	BCL2, CD10, MUM1	BCL2 ≥ 50% and CD10 or BCL6 ≥ 30%
Nyman [26]	MUM1, FOXP1	≥30% staining to be considered positive

**Table 2:** Diagnostic approaches to diffuse large B-cell lymphoma with comparative advantages and disadvantages to each method.

Diagnostic Technology	Advantages	Disadvantages	Subclassification Methods/Key Studies
Immunohistochemistry	Fast turnaround time Cost effective Antibodies readily available Hans classifier adopted by the WHO	Decreased sensitivity and specificity	Hans classifier (Hans et al. 2004)
Gene expression profiling	Increased sensitivity and specificity	Decreased turnaround time Increased expense Accessibility in routine diagnostic setting Not mandatory for WHO diagnosis	Cell of Origin (Alizadeh et al. 2000)
Cytogenetics and fluorescence in-situ hybridization (FISH)	Rapid turnaround time	Increased expense Requires a separate cytogenetics lab	Exclude high-grade B cell lymphoma with MYC and BCL2 and/or BCL6 rearrangements
Next generation sequencing	Increased sensitivity and specificity Identify genetic subgroups and potential new therapeutic options	Increased cost Increased turnaround time	Clusters C1–C5 (Chapuy et al. 2018) MCD, N1, BN2, and EZB (Schmitz et al. 2018) Stromal 1 and 2 signatures of the microenvironment (Lenz et al. 2008; Cioronianu et al. 2019)

## Data Availability

Not applicable.
